# Eat to Treat: The Methods and Assessments of a Culinary Medicine Seminar for Future Physicians and Practicing Clinicians

**DOI:** 10.3390/nu15224819

**Published:** 2023-11-17

**Authors:** Kate Donovan, Olivia W. Thomas, Ty Sweeney, Tyler J. Ryan, Sonja Kytomaa, Molly Zhao, Wayne Zhong, Michelle Long, Iniya Rajendran, Suzanne Sarfaty, Carine Lenders

**Affiliations:** 1 Department of Pediatrics, Boston Medical Center, Boston, MA 02218, USA; 2 Department of Food and Nutrition, Boston Medical Center, Boston, MA 02218, USA; 3 Department of Medicine, Chobanian & Avedisian School of Medicine, Boston University, Boston, MA 02218, USA; 4 Department of Medicine, Johns Hopkins University School of Medicine, Baltimore, MD 21224, USA; 5 Department of Medicine, University of Massachusetts Chan Medical School, Worcester, MA 01655, USA; 6 Section of Gastroenterology, Boston Medical Center, Chobanian & Avedisian School of Medicine, Boston University, Boston, MA 02118, USA; 7 Department of General Internal Medicine, Boston Medical Center, Chobanian & Avedisian School of Medicine, Boston University, Boston, MA 02118, USA; 8 Department of Pediatrics, Pediatric Nutrition and Fitness for Life, Boston Medical Center, Chobanian & Avedisian School of Medicine, Boston University, Boston, MA 02118, USA

**Keywords:** nutrition, education, culinary medicine, registered dietician, medical student, kitchen, cooking, social determinants of health

## Abstract

Nutrition-associated chronic disease is an epidemic in the United States (US), yet most medical schools lack adequate nutrition education. We developed a six-session culinary medicine (CM) seminar entitled “Eat to Treat: A Nutrition Course for Future Clinicians” that teaches culinary skills, nutrition science, and counseling techniques to improve clinical nutrition management. The seminar was offered in-person to first-year medical students in a medical school-based teaching kitchen from 2017 to 2019. A virtual three-session course was also offered to practicing clinicians in 2020. Voluntary self-efficacy questionnaires were collected at the beginning of the first and last sessions of the student seminar, and paired *t*-tests determined the course’s effect on survey items. A total of 53 first-year medical students attended the program over five semesters, and 39 students (73.6%) completed both surveys. All except one measure of self-efficacy were significantly higher at session 6 than session 1 (*p* < 0.05). A post-course survey was utilized for the clinician seminar and of the 31 participants, 14 completed the surveys; 93% and 86% of respondents agreed the course was clinically relevant and improved their confidence, respectively. We developed a CM curriculum that improved nutrition knowledge and confidence among a professionally diverse cohort and may represent a scalable education model to improve nutrition education in US medical schools.

## 1. Introduction

Chronic diseases impose substantial health and financial burdens in the United States [1]. Poor nutrition is a modifiable risk factor and major contributor to many preventable chronic diseases such as hypertension, diabetes, and obesity [2]. While nutrition counseling is considered a primary intervention and should be included in routine visits, only one in five patients with chronic diseases receives nutrition counseling [3]. This disconnect is likely driven by several factors, as physicians report lacking the skills, knowledge, clinical time, financial incentive, and confidence to effectively counsel patients on important dietary changes [4]. Inadequate nutrition education in medical schools may account for physician-reported lack of confidence in counseling patients on nutrition [5]. Nutrition education in medical schools is lacking both in the US and abroad [6]. A 2014 survey of US medical schools found that only 27% of respondents met the recommended minimum of 25 h of nutrition education set by the National Academy of Sciences. Furthermore, 36% of respondents provided less than half the recommended hours [7]. In addition to a lack of physician training in nutrition, there is an underutilization of registered dietitians (RDs), who are trained in administering medical nutrition therapy [8].

To address the lack of nutrition training in medical school and the underutilization of registered dieticians in clinician practice, initiatives seeking to enhance nutrition education have emerged in both medical schools and continuing education courses. Culinary medicine (CM) is an innovative and emerging approach that has become a recognized method of teaching practical skills [9,10]. It integrates food planning, preparation, and cooking with health sciences to improve health outcomes. Hands-on CM training has been well received by both students and instructors and has become recognized by professional societies for its ability to promote health and wellbeing [11]. For instance, the Mediterranean-style diet and Diet Approaches to Stop Hypertension have been shown to improve glucose metabolism, blood pressure, lipid metabolism and other measures of cardiometabolic health among people with diabetes or cardiovascular disease [12]. While internally validated curricula and opportunities for licensure already exist—such as Tulane University’s Goldring Center for Culinary Medicine curriculum licensed to 12 medical schools nationwide—many institutions prefer to construct their own personalized programming tailored to student scheduling, institutional resources, and patient needs [13,14]. Although many programs are similar, best practices and effective dose have yet to be determined [15]. 

Starting in 2006, a novel student-centered model of nutrition medicine education was developed at Boston University School of Medicine (BUSM) [16,17]. These efforts have been shared nationally; as a result, BUSM has been ranked in the top 10% of medical schools nationally for the number of hours with nutrition content [18,19,20]. Yet, medical students at BUSM continue to feel underprepared to counsel patients in nutrition and provide referrals. The BUSM medical students scored on average 52% on a nutrition knowledge assessment and reported low confidence in providing dietary counseling (55%), referring patients to registered dietitians (38%), and connecting patients to food resource programs (24%) [7,21]. Following initial Test Kitchen efforts (now the Boston Medical Center (BMC) Teaching Kitchen) and in response to the need for increased practical food and nutrition skill education at our institution, we developed a CM seminar series entitled “Eat to Treat: A Nutrition Course for Future Physicians and Practicing Clinicians”, or ETT for short. This seminar is designed to equip providers with a deeper understanding of nutrition and diet and their impact on chronic disease. The course is intended to bridge gaps in nutrition knowledge and teach practical skills such as sourcing affordable ingredients, incorporating fruits, vegetables, whole grains, and lean meats into common meals, and adapting recipes to meet the needs of patients with limited resources and nutrition-related chronic diseases. This seminar series combines hands-on culinary training, didactic sessions, and patient simulations under the supervision of an RD. After 3 years and six cohorts, we expanded the program to offer a pilot series for practicing clinicians. We hypothesized that this program would increase medical student and provider confidence in practical nutrition and counseling skills and build confidence in the utilization of RD referrals. 

## 2. Materials and Methods

### 2.1. Study Design 

To understand the acceptance and impact of ETT, exploratory and descriptive surveys were used for evaluation and self-reported outcomes. A pre-post survey design was used for the medical student group. A post survey was used for the clinician group as part of the continuing education credit process. Informed consent was received from all participants. The study was approved by the Boston University Independent Review Board (IRB). 

### 2.2. Recruitment 

Students were recruited for ETT by student ambassadors, upper-class students who have previously participated in the seminar. These students are responsible for recruitment and mentorship during the seminar. Clinicians were recruited through flyers, email marketing, and frequent postings in the employee newsletter. Registration was coordinated through Boston University’s Continuing Education Department. A standard fee was charged for those receiving six continuing education credits including Continuing Medical Education (CME), Continuing Nursing Education (CNE), and Continuing Education Units for Registered Dietitians (CEUs). The course was offered free to those not receiving credit and to trainees.

### 2.3. Program Description 

ETT is a seminar designed to equip providers with a deeper understanding of food and nutrition and their impact on chronic disease. Additionally, it aims to help participants enhance their communication and counseling skills to better engage with and treat their patients, while understanding the challenges faced by many patients when attempting to achieve or maintain a healthy lifestyle. The program seeks to support these objectives through three learning domains:

1. Nutrition knowledge: Participants will gain a better understanding of basic nutrition and medical nutrition therapy as they relate to common nutrition-related chronic diseases.
2. Nutrition counseling: Participants will acquire specific skills to effectively counsel patients on food and nutrition as well as to address social determinants of health (SDoH) in culturally sensitive ways. Additionally, participants will gain an understanding of the role of the RD as a part of the care team and will learn how and when to refer to RDs and to other nutrition resources.
3. Culinary skills: Participants will learn basic culinary skills to enhance their practical understanding of nutritional interventions and to better relate to their future patients.

ETT originated as a six-session nutrition seminar offered to first year medical students at BUSM. Participation was voluntary and class size was capped at 16 students due to space limitations. The course was first offered in the fall of 2017 and has continued to be offered since its inception in a modified format. 

The first five 2-h sessions integrate the three domain objectives described above (nutrition knowledge, nutrition counseling, and culinary skills) into lessons that cover introduction to nutrition, food insecurity, obesity, hypertension, and diabetes mellitus (Table 1). The seminar is offered in the BMC Teaching Kitchen under the supervision of an RD. This kitchen provides access to a hands-on culinary learning experience wherein students prepare a meal based on the class topic. Throughout the cooking process, the RD provides a brief nutrition lecture about the session topics. The group then comes together to share the meal while learning counseling skills via a mini-case study or clinical vignette. 

In the final session, participants are split into three teams for an “Iron Chef” style competition in which they are assigned a disease state previously covered, a protein that is available in the BMC food pantry, and a single heat source that simulates constraints in access to kitchen appliances (i.e., microwave, oven, or stovetop). Students are tasked with creating a healthy, disease-appropriate meal and explaining why their meal would be appropriate for a patient with the assigned disease.

ETT for students is supported by student ambassadors. Each year, the group meets to evaluate the seminar’s efficacy and to update the curriculum. Supplies and funding for the instructor are provided by the BMC Teaching Kitchen and Boston University Chobanian & Avedisian School of Medicine Office of Enrichment. Additional food resources are currently provided by the BMC Preventive Food Pantry and BMC Rooftop Farm. However, a $10 participation fee was required in the first two semesters to cover food and supply costs prior to institutional funding being available. The course objectives are as follows:

1. Improve the students’ self-perceived health practice.
2. Improve the students’ self-efficacy in nutrition assessment and management including:
  a. Confidence to assess patients’ diet and nutrition,
  b. Confidence to communicate with and counsel patients on food and nutrition-related diseases,
  c. Confidence to communicate and direct to resources addressing social determinants of health (SDoH) as they relate to food and nutrition,
  d. Confidence to understand scope of RD practice and interprofessional team roles in management of nutrition-related diseases.

### 2.4. Practicing Clinician and COVID-19 Pivot 

An adapted version of this course was offered to practicing clinicians in 2020. The six-part series was condensed into a three-classes format and the content was revised to meet the needs of more highly trained participants. Due to the COVID-19 pandemic, the clinician course was administered virtually. Classes included a 90-min didactic lecture and a 30-min cooking demonstration. Content for the course was reviewed by an interprofessional advisory board including physicians, dietitians, nurses, investigators, and clinical leaders from cardiology, endocrinology, gastroenterology, family medicine, internal medicine, and obesity medicine. We gathered additional input from a trainee perspective from residents, medical and dietetic students. The design and curriculum expanded on the results from the student seminar series. 

An electronic course manual was distributed to all participants prior to the start of the course. We encouraged participants to use the manual as a reference guide for both the course curriculum and recipes. The lectures were delivered by researchers, physicians, and registered dietitians employed at the academic medical center as well as outside experts. The adjusted course objectives were as follows:

1. To evaluate the acceptability of the course among practicing clinicians,
2. To improve self-efficacy in talking about and addressing nutrition with patients,
3. To inform practice and inspire practice implications.

### 2.5. Data Collection Measures 

Student data for this study were collected over 3 years (2017–2019). Self-efficacy questionnaires were administered at the beginning of the first and last sessions. Results from the questionnaires were captured via a Likert scale (1–5) with ‘1’ being ‘Not Confident’ and ‘5’ being ‘Very Confident’. Students were also asked to rate their personal diet/nutrition and exercise habits on a scale from 1 to 10 with ‘1’ being ‘Very Unhealthy’ and ‘10’ being ‘Very Healthy.’ Surveys were voluntary and were not required for enrollment in the seminar.

Clinician data were only collected after the pilot series (2020) through a post-class survey and evaluation. Both students and clinician participants from both the student and clinician courses were solicited for suggestions on how to improve the Eat to Treat curriculum after the final session. In order to generate themes, study authors blinded to subject identity reviewed qualitative data and assigned codes to each item as they related to the survey question. Codes were then reviewed and grouped into themes that captured overall patterns. After refining the themes, representative examples were chosen.

### 2.6. Statistical Analysis 

The student data were analyzed using a paired sample *t*-test to determine whether participants’ confidence in accomplishing specific tasks changed significantly after completion of the course. No statistical analysis was used to analyze the clinician data.

## 3. Results

### 3.1. Student Seminar

A total of 53 first year medical students attended the program over five semesters from the fall of 2017 through the fall of 2019. A total of 39 students (73.6%) completed the self-efficacy questionnaire at the beginning and end of the course and responded to a questionnaire on satisfaction and plan to change their practice. Except for the trainees’ self-perceived health practice, all measures of self-efficacy in nutrition assessment—including confidence in assessing and providing counseling on patients’ diet and nutrition, incorporating SDoH in counseling, and recruiting RDs for management—were significantly higher at session 6 than at session 1 (*p* < 0.05, Table 2). All respondents reported that they would recommend this seminar to future students.

### 3.2. Clinician Seminar 

There were 31 participants in the clinician seminar. A total of 14 participants registered for continuing education credit (3 dietitians, 5 physicians, and 6 nurses), 10 participants were trainees and 7 were providers who did not register for credit. Only those participants claiming credit responded to our post-course survey. The general acceptance of the course was assessed and is represented in Table 3. Most respondents (*n* = 13) felt that the information was relevant to their practice and all respondents (*n* = 14) felt that the course effectively demonstrated the clinical function of CM. Most respondents also reported increased confidence in employing CM into their practice either through counseling, referrals, or shared medical appointments (*n* = 12). 

### 3.3. Course Feedback

Qualitative feedback was also collected in the questionnaire to help better understand the experience of participants. Feedback was divided into two categories: “what worked best” and “what could be improved”. “What worked best” themes that emerged included satisfaction with the session structure and the interactive nature of the sessions. Specifically, participants enjoyed the flow of the sessions, the format (three 2-h sessions) and the virtual offerings (Table 3). “What could be improved” themes that emerged included offering more interactive activities and shifting content to include a lesser focus on scientific evidence and greater focus on practical solutions (Table 4). The last area assessed was participants’ intended changes after taking the course. Emerging themes included changes to personal diet, patient care, and utilization of an interdisciplinary team and nutrition resources (Table 5).

## 4. Discussion

The CM curriculum in this study increased measures of self-efficacy among medical students and was well received by both students and clinicians. Though new programs are emerging, nutrition and culinary education are still not widespread or commonly available at academic medical centers [10]. Since the creation of our program, other CM curricula have been developed with both in-person and/or virtual experiential learning components aiming to improve physician nutrition counseling. Many of these programs offer didactic-based online modules [22,23], while others offer in-person training [24]. There is a similarly wide spectrum in terms of target audience, with different programs catering to learners across the educational timeline from medical students to residents, to fellows, and beyond [25]. A review identifying 12 published CM curriculums suggested that these programs—most of which relied on pre- and post-intervention surveys and open-ended qualitative questionnaires—consistently demonstrated improvement in reported student knowledge [10]. However, our program provides several unique elements. We leverage the expertise of RDs, emphasize hands-on learning opportunities, and provide training to participants across the medical training spectrum. Furthermore, given our program is positioned within a safety-net hospital, strategies to address culturally diverse and underserved communities through effective and thoughtful interventions is foundational to the curriculum. Food insecurity and social determinants of health were not explicit focuses of previously published programs [10]. 

RDs were major contributors to the planning and facilitation of the programs, further adding to the body of literature that suggests RDs are effective and essential to CM training [26]. In addition, learning about the RD referral process was very well received by both the medical students and the clinician group participants. As described by the Academy of Nutrition and Dietetics (AND), there is a continued need for improving referrals to RDs, especially as medical nutrition therapy offered by RDs has been shown to be valuable in reaching patient health goals such as weight loss and has been found to be cost-effective [27,28]. In fact, nutrition experts believe that referring patients to RDs is one of the most important factors contributing to sustained behavior change [27]. 

Several limitations to this study should be noted. First, participation was voluntary and therefore self-selection of students and providers with an affinity for nutrition education likely biases the results. Demographic data including age, sex, professional degrees, and socioeconomic status among course participants were not collected. Second, the survey has not been validated at the time of writing, though questions were adapted from previously validated surveys. Third, the questions assessed only self-reported data as opposed to objective measures. Fourth, due to the COVID-19 pandemic, the clinician course was only able to be offered virtually and the experiential learning component was minimal. The course was also truncated to three sessions. This could be addressed with breakout sessions for skills practice in future virtual courses. In addition, in-person or hybrid sessions for clinicians could also be offered. Fifth, the clinician course pooled data from all providers and did not assess how professional degrees and backgrounds might influence course efficacy. Lastly, attrition in the student course was likely due to a combination of factors including student absence at the time the survey was administered at the end of the sixth course and failure to fill in the personal identifier item on the survey. The comparatively low survey response in the clinical course was likely due to a lack of accountability in a virtual format.

Those wishing to integrate CM into medical training should be mindful of certain obstacles to implementation. In particular, the culinary skills portion of this curriculum requires a cooking space outfitted with conventional kitchen supplies and a staff member with the training to teach these skills. As not all potential consumers may have access to a teaching kitchen, this may limit adoption, though other institutions have found creative solutions to this potential barrier [22]. Similarly, if a cooking space is available, its size would limit the number of participants at one time. Finally, funding is a principal barrier to program longevity [10]. The procurement of ingredients, renting of teaching space, and compensation for instructors would require funding sources that may not be available at all institutions. Given these limitations, lower-resourced options such as live-stream virtual classes and/or on-demand pre-recorded videos that optimize the participants’ home kitchen may be more feasible. 

We have demonstrated that CM education can be effectively delivered to medical students and clinicians, with participants enjoying the format and content and reporting improvement in their knowledge and self-efficacy. Our findings suggest that similar courses may help bridge the gap in nutrition medicine knowledge and confidence reported by practicing physicians nationally. However, there are insufficient data to optimize CM training nationally. For instance, significant heterogeneity in course time exists across institutions. The results from this study suggest that the course significantly improved student self-efficacy over 12 h of instruction, while other programs may include up to 28 h of instruction or more to accomplish their respective goals [29]. Further studies should include objective measures of changes in CM knowledge and explore the time necessary to achieve this knowledge. Additionally, prospective studies looking at the impact of such sessions on practice would be useful. 

## 5. Conclusions

This CM curriculum is one method by which the issue of inadequate nutrition education among physicians may be addressed. The content of our programs will continue to evolve as nutritional science changes and incorporate participant feedback and inter-institutional collaboration. We hope that other institutions will leverage our framework, integrating CM into their own medical training to better address the dearth of nutrition education in US medical schools. By improving the delivery of CM concepts to medical trainees, future physicians may be better equipped to use food and nutrition to help alleviate the burden of chronic disease in the U.S.

## Figures and Tables

**Table 1:** Eat To Treat six-session course outline.

Nutrition Topic	Kitchen Skill	Recipe	Counseling Skill
Intro to nutrition and the Harvard Plate	Knife skills, food safety, and methods of cooking vegetables	Balancing your plate with personal pizzas	Assessing diet quality using the 24-h Diet Recall and the Food Frequency Questionnaire
The impact of social determents of health on diet quality	Methods of cooking plant and animal proteins	How to use rotisserie chicken, canned beans, and roast a sheet pan dinner	Benefits of an interprofessional team and tactics to screen and refer patients to dietitians, community-based organizations, and food assistance programs/medically tailored meals
Weight management and obesity	Methods of cooking grains	Rice, quinoa, and farro grain bowls with vegetables and homemade dressings	Practicing empathy and avoiding stigmatization when counseling patients on weight management
Hypertension	Weights & measurements of ingredients	Yogurt parfaits, overnight oats, and oat bites	Strategies to communicate disease severity to activate patients
Type 2 diabetes	Methods of cooking eggs	Muffin tin omelets and shakshuka	Using SMART goals and motivational interviewing to enhance behavior change
Final Session	Apply knowledge through the Iron Chef Competition		

**Table 2:** Student self-efficacy questionnaire responses from baseline (*n* = 53) to course completion (*n* = 39).

#	Assessment Categories	Question	Answer Scale	Mean Pre-Course Score	Mean Post-Course Score	*p*-Value
1	1	How would you rate your personal food, nutrition, and exercise habits	1–10	6.5	6.9	0.06
Questions 2–13 ask participants “how confident do you feel in your ability to accomplish the following tasks?”						
2	2.2	Communicate with patients	1–5	3.6	4.0	0.002 *
3	2.1	Assess a patient’s food intake	1–5	2.8	4.2	<0.001 *
4	2.2	Explain the role of nutrition in disease prevention and treatment (ex: obesity, hypertension, diabetes)	1–5	3.0	4.0	<0.001 *
5	2.2	Explain disease severity to patients	1–5	2.8	3.8	<0.001 *
6	2.2	Counsel patients about dietary changes	1–5	2.8	3.8	<0.001 *
7	2.3	Direct patients to appropriate nutrition resources	1–5	2.5	3.8	<0.001 *
8	2.3	Talk to patients about food security and refer them to appropriate food and nutrition programs (SNAP, WIC, food pantries, etc.)	1–5	2.4	3.9	<0.001 *
9	1	Prepare a healthy meal for four people for $10 or less	1–5	2.7	4.1	<0.001 *
10	2.4	Understand the role of registered dietitians and when to refer patients to them for further care	1–5	2.7	4.6	<0.001 *
11	2.2	Stimulate health behavior change in a patient	1–5	2.6	3.8	<0.001 *
12	2.2	Set achievable goals for health behaviors with patients	1–5	2.9	4.3	<0.001 *
13	2.2	Respect cultural beliefs/practices when counseling a patient about nutrition (ex: traditional diets, religious beliefs, etc.)	1–5	3.4	4.4	<0.001 *

* Indicates statistical significance with *p* < 0.05.

**Table 3:** General course acceptance among clinician course participants.

Survey Response	43.8% (*n* = 14/32 Participants)
How would you rate this activity overall?	(5 = excellent, 1 = poor, please circle one)
	Mean: 4.4
The educational content was presented objectively and was free of commercial bias	Yes—100%
	(*n* = 14)
Do you feel that the information in this activity was based on the best evidence available?	Yes—100%
	(*n* = 14)
Do you feel that the information in this activity was relevant to your practice?	Yes—92% (*n* = 13)
	No—0%
	Partially—8% (*n* = 1)
Do you plan to make any changes based on what you learned in this activity?	Yes—86% (*n* = 12)
	No—14% (*n* = 2)

**Table 4:** Provider self-efficacy questionnaire after course completion (*n* = 14).

As a Result of This Activity, Do You Feel You Are Better Able to	Yes	No	Partially	No Response
Understand the function of culinary medicine in a hospital setting with 80% accuracy	100%	0%	0%	0%
List at least two real food and cultural examples of the Plate Method	92%	0%	8%	0%
Identify the three therapeutic diets reviewed for the treatment of metabolic disease symptoms and the clinical criteria and nutrition considerations for each with 80% accuracy	79%	0%	21%	0%
Increase consideration of patients’ access to food, and be able to provide at least two feasible and affordable food solutions	92%	0%	8%	0%
Identify three ways to introduce culinary medicine into practice, either through counseling, referrals, or shared medical appointments	86%	0%	8%	8%
Identify two strategies to empower patients to increase home cooking	92%	0%	8%	0%

**Table 5:** Provider’s intended practice implications after course completion (*n* = 14).

Personal Diet
“I will start to change the way I look at portions”
“I started using avocado oil for high temperature situations instead of olive oil”
“I made a noodle soup with brown rice noodles the other day and made zucchini noodles per [the instructors’] suggestion and it was great!”
Patient Care
“Bring more cultural awareness to the types of foods I discuss with patients and incorporate the foods they are already eating”
“Inquire and listen to patients about their hopes, motivations, preferences, and obstacles to eating well”
“I have become more mindful in the way I communicate with others about food and weight loss. I will now strategize to reinforce more positivity when having difficult conversations with people around food”
“I will use these nutrition tips for my general internal med practice”
Utilize Interdisciplinary Team and Nutrition Resources
“More direct referrals to nutrition and utilizing teaching kitchen”
“I am a new RD, I feel it’s important for me to learn more from the experts who have more experience providing care”

## Data Availability

The data presented in this study are available on request from the corresponding author. The data are not publicly available to maintain privacy.
